# Inactivation of Inulinase and Marination of High-Quality Jerusalem Artichoke (*Helianthus tuberosus* L.) Pickles With Screened Dominant Strains

**DOI:** 10.3389/fbioe.2020.626861

**Published:** 2021-01-20

**Authors:** Li Zhang, Wei Liu, Jiahong Ji, Lina Deng, Qian Feng, Wujian Shi, Jian Gao

**Affiliations:** School of Marine and Bioengineering, Yancheng Institute of Technology, Yancheng, China

**Keywords:** Jerusalem artichoke pickles, inulinase, enzyme inactivation, microbial powder, inulin

## Abstract

Freshly harvested Jerusalem artichoke tubers contain inulinase, an enzyme that requires inactivation, because of its ability to hydrolysis inulin into fructose, which can be consumed by microorganism during marination. As the traditional pickling process takes 6 months, and involves the addition of a large amount of salt (18–20%), this production strategy is uneconomical and increases the nitrite intake. Additionally, miscellaneous bacteria produced during pickling affect the product taste. In this study, the enzyme inactivation effects of NaCl, NaHCO_3_, and ultrasound were evaluated. NaHCO_3_ treatment results in the highest degree of enzyme inactivation; however, the quality and flavor of the obtained Jerusalem artichoke pickles were not ideal. The Jerusalem artichoke pickles in which the enzymes were inactivated using a combination of NaCl and ultrasound exhibited better flavor than those exposed to NaHCO_3_; further, this combination reduced the inulinase activity of the Jerusalem artichokes to 2.50 U/mL, and maintained the inulin content at 61.22%. The strains LS3 and YS2, identified as *Enterococcus faecalis* and the salt-tolerant yeast *Meyerozyma guilliermondii*, respectively, were the dominant microorganisms isolated from the pickle juice. Jerusalem artichokes with inactivated inulinase were pickled with microbial powder, separated, purified, and dried to remove the natural Jerusalem artichoke sauce. This process shortened the fermentation cycle and improved product quality.

## Introduction

The Jerusalem artichoke, also known as *Helianthus tuberosus* L., belongs to the *Compositae* family and the sunflower genus. The artichokes are widely cultivated and distributed, owing to their ecological adaptability and fertility under harsh conditions, such as salinity, alkalinity, cold, drought, and wind (Shao et al., 2019). As a non-grain raw material that has a high yield as well as soil-improving and environment-beautifying attributes, the Jerusalem artichoke can provide many ecological and economic benefits (Guo et al., 2018). Jerusalem artichoke tubers have a fine texture, a crisp, sweet taste, and they are rich in nutrients, including inulin, vitamins, minerals, amino acids, and trace elements (Guo et al., 2018; Judprasong et al., 2018). Importantly, the Jerusalem artichokes serves as an ideal raw material for high-quality pickles (Lv et al., 2019).

Pickled vegetables undergo a traditional microbial fermentation process that employs the preservative effect of salt to prolong the shelf life of the vegetable. The popularity of pickled vegetables has been steadily rising, owing to their unique color, aroma, and low cost (Behera et al., 2020). For instance, the unique sensory properties (i.e., flavor and mouthfeel) and the potential health benefits associated with lactic acid fermentation in the Paojiao produced in Yunnan—considered the most authentic—have made it popular in China (Ye et al., 2020). Another pickled product, Kimchi is world-renowned for being rich in vitamin A, thiamine, riboflavin, calcium, iron, and lactic acid-producing bacteria. In addition, several studies have reported that fermented soybean products possess beneficial properties, including antioxidant, antimicrobial, blood pressure-lowering, and antidiabetic activity (Hwang et al., 2017).

**Figure 1:**
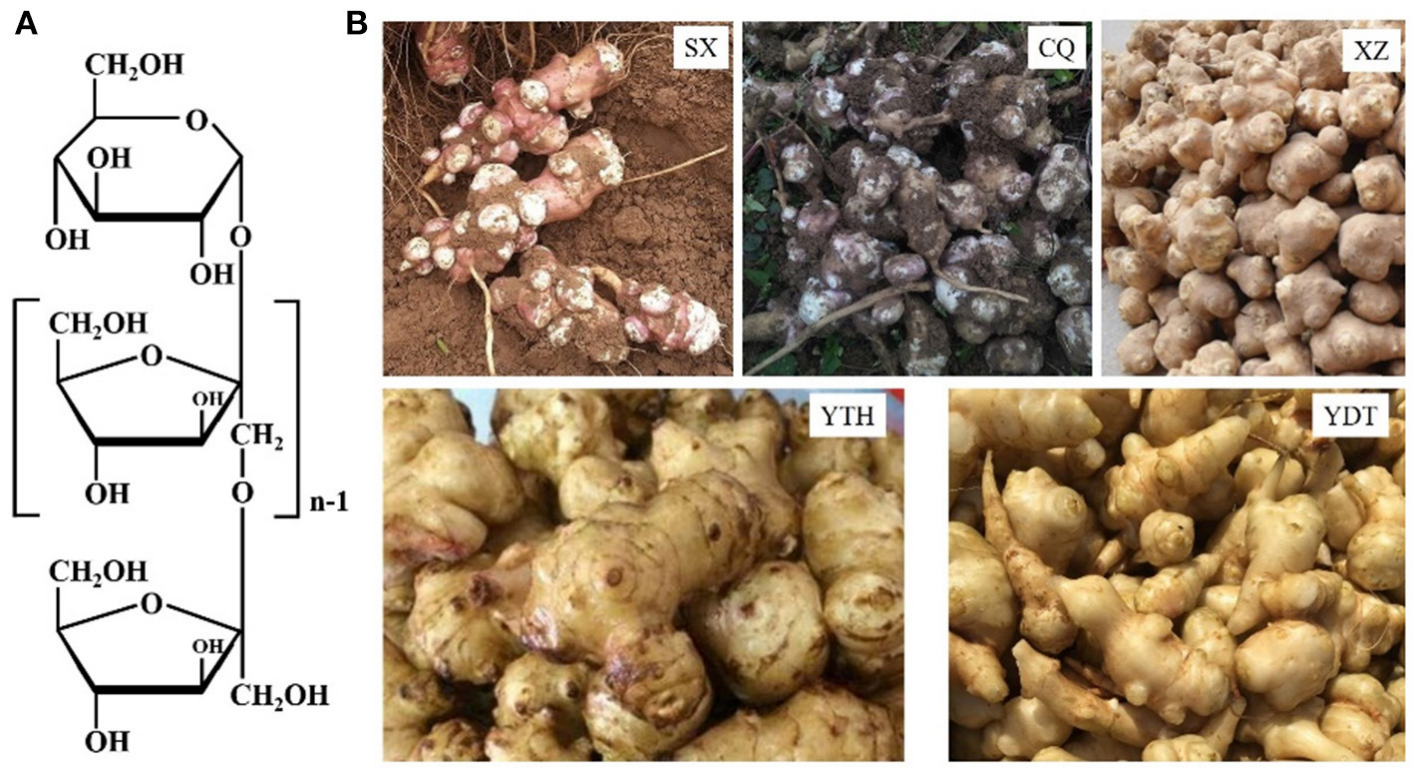
The chemical structure of inulin **(A)** and Jerusalem artichoke tubers **(B)** harvested from SX, CQ, XZ, YTH, and YDT.

Jerusalem artichoke pickles are crisp, fragrant, slightly sweet, and tender; further, they are easy to store, which is why they are favored by consumers. Healthier and low-salt pickles are being prepared to match the improved living standards. Moreover, Jerusalem artichoke tubers are rich in inulin, which is the second most-common plant storage carbohydrate after starch and accounts for ~50–70% of the Jerusalem artichoke tuber stem weight (Rubel et al., 2018). Inulin is a linear polysaccharide in which D-fructofurans are linked by a β (1 → 2) bond, with a D-glucose residue—typically residing at the end of individual fructose chains—being linked to fructose by an α (1 → 2) bond (Zhu et al., 2020). The average molecular weight of inulin is ~5,500 Da, and the molecular formula is GFn, as shown in Figure 1, where G represents the terminal glucose, F represents fructose, and *n* represents the number of fructose units. Inulin is used as a healthcare product as it has been shown to regulate blood sugar, reduce fat and weight, improve the intestinal environment, promote mineral absorption, and prevent constipation (Shoaib et al., 2016; Wang et al., 2016; Yu et al., 2018). Thus, the development of Jerusalem artichoke pickled products is of economic importance; however, the enzymes involved in the pickling process may alter the flavor and texture of the product.

One problem that is encountered during the picking of Jerusalem artichokes is the presence of inulinase. During the pickling of fresh Jerusalem artichokes, inulin present in these vegetables can be acted on by inulinase. Inulinase can hydrolyze the β (1 → 2) glycoside bonds between the fructose moieties of inulin, and this process is widely used in the production of oligosaccharides and high-fructose syrup (Singh et al., 2020). Inulin can be degraded by inulinase into fructose, which is consumed by microorganism during pickling. Furthermore, inulinase can alter the quality of pickled products, so it is necessary to take appropriate measures to check inulinase activity in Jerusalem artichoke pickles during the curing process. This not only preserves the beneficial health properties of inulin, but also protects Jerusalem artichokes from putrefaction and deterioration, and inhibits discoloration, flavor change, and nutrient content reduction due to enzyme activity (Makroo et al., 2020).

Another problem in the processing of Jerusalem artichoke pickles is the long curing period, during which a large amount of salt is added (18–20%) and the pickles are exposed to an environment with high nitrite levels and infectious microbes. Notably, the procedure is uneconomical, and the high levels of nitrite and miscellaneous microorganisms produced during the pickling process affect the taste of the product. In this study, dry powders of lactic acid bacteria and yeast strains were separated from pickle juice samples that had been obtained from a pickle factory and used as experimental strains. Microorganisms (mainly lactic acid bacteria and yeasts) play a pivotal role in pickling and affect the quality and safety of the final product (Behera et al., 2020). The inulin and nitrite contents of the Jerusalem artichokes subjected to traditional and improved pickling processes were determined, with the goal of screening and identifying naturally-brewed strains and shortening the pickling period.

Inulinase inactivation in Jerusalem artichoke pickles presents many challenges, including the prevention of inulin degradation and preservation of the taste of pickles, while ensuring the production of high-quality Jerusalem artichoke pickles. To our knowledge, no method has been developed to inactivate the inulinase—to ensure inulin preservation—during the production of Jerusalem artichoke pickles. Therefore, it is necessary to prepare high-quality pickles with the raw material of Jerusalem artichoke tubers and explore inulinase inactivation, so as to maintain the inulin content of Jerusalem artichokes during curing, thereby preserving the health benefits associated with artichokes. Specifically, the aim of this study was to investigate the effects of enzyme inactivation during the pickling of Jerusalem artichokes. The effect of three different treatments, i.e., NaCl, NaHCO_3_, and ultrasound on inulinase inactivation during Jerusalem artichokes pickling were evaluated; their effects on the sensory qualities of the pickles were studied. Then, Jerusalem artichokes with inactivated inulinase were pickled using microbial powder, which had been separated, purified, and dried from the natural Jerusalem artichoke sauce. This process shortened the fermentation cycle and improved the product quality. Efforts are underway to develop nutritious, therapeutic, low salt, and naturally preserved vegetables.

## Materials and Methods

### Raw Materials and Crude Inulin Extract

Jerusalem artichoke tubers that were fresh, shiny, even in size, and free from decay and mechanical damage were purchased from Shan Xi (SX), Chong Qing (CQ), Xu Zhou (XZ), Yancheng Ting Hu (YTH), and Yancheng Dong Tai (YDT). The tubers were washed, dried, peeled, and weighed. Inulinase activity was determined by preparing inulinase crude extract from 10 g of fresh Jerusalem artichoke tuber. The remaining Jerusalem artichoke tubers were cut into thin slices and then heated in boiling water at 100°C for 5–10 min to inactivate polyphenol oxidase (PPO), which is responsible for browning in most vegetables (Makroo et al., 2020). Then, the artichoke slices were dried in an oven at constant temperature, and the weight was recorded. Jerusalem artichoke powder (ground using a grinder and then sifted through a 40-mesh sieve) was mixed with distilled water at a ratio of 1:8 and incubated in a 70°C water bath for 2 h. The pH was adjusted to 10.0 with lime milk, and the solution was then incubated in an 80°C water bath for 1 h. Inulin extract was obtained after removing the filter residue using eight layers of gauze. The total sugar and reducing sugar content were determined to calculate the inulin content of the Jerusalem artichoke tubers.

### Inulinase Inactivation

#### Salt Stress

Fresh Jerusalem artichoke tubers were washed, dried, sliced into 5 mm-thick slices weighing 100 g, placed in 600 mL pickle jars, and salted with NaCl. The fresh Jerusalem artichoke tubers contained ~80% water. After salt was added, the Jerusalem artichoke slices exuded water to dissolve the salt, and the exudate covered all of the pickled Jerusalem artichoke slices. The taste of Jerusalem artichoke pickles can change in the presence of excessive salt and nitrite, and the traditional pickle marinade of 18–20% (w/w) salt is not healthy. Therefore, the concentration of salt was reduced to 10–16% (w/w) in this study, which is cost-effective and benefits the green economy. Jerusalem artichoke slices (10 g) pretreated with salt were blended with 50 mL chilled phosphate buffer. The supernatant was collected after centrifugation at 5,000 r/min for 10 min and treated as a crude enzyme to determine inulinase activity.

#### NaHCO_3_ Treatment

Fresh Jerusalem artichoke tubers were washed, dried, sliced into 5 mm thick slices weighing 100 g, placed in 600 mL pickle jars, and salted with NaHCO_3_. The concentration of NaHCO_3_ was reduced to 10–16% (w/w), and inulin content and inulinase activity were determined.

#### Ultrasonic Treatment

Jerusalem artichoke slices (100 g) were weighed accurately and then layered in a 500 mL beaker containing 150 mL distilled water. Then, inulinase was inactivated in an ultrasonic cleaning machine at room temperature with the following parameters: ultrasonic frequency, 40 KHz; ultrasonic power, 480 W for 20–70 min; gradient, 10 min. Inulin content and inulinase activity were then determined.

### Microbial Powder Preparation

#### Screening for Lactobacillus in Pickle Juice

The Jerusalem artichoke pickle juice was diluted to the optimum concentration and plated onto LB agar medium. After incubating at 30°C for 2–3 d, bacteria with different growth potentials were selected and cultured repeatedly on LB medium, and pure bacteria were obtained. The pure bacteria were stored in 30% glycerin in a −20°C refrigerator for later use. The preserved strains were lined up and cultured in sterilized MRS solid medium, at 37°C for 3–4 d. Bacteria that were able to grow on MRS medium and exhibited melt-calcium rings were observed and recorded. The pure bacteria that grew on MRS culture medium were Gram-stained and observed using oil immersion microscopy (Zheng et al., 2020). The gram-positive bacteria were suspected to be *Lactobacillus* strains and were cultured on an inclined plane and preserved. *Lactobacillus* strains were identified by 16S rDNA sequencing (Won et al., 2020).

Saccharide fermentation tests were conducted as follows. Saccharide fermentation tubes were prepared, and inverted tubules were inserted to identify whether gas was generated. The tubes were marked and sterilized. The name of the fermentation medium and the number of lactic acid bacteria to be inoculated were marked on the outer wall of each tube with a marker pen. Different *Lactobacillus* strains were inoculated into corresponding labeled sugar fermentation tubes, which were shaken gently to blend and prevent bubble generation in the inverted tubes. An uninoculated sugar fermentation tube was used as a blank control. The sugar fermentation tubes and blanks were inoculated for 24–48 h at 37°C. The color change in each test tube was examined to determine whether bubbles had formed in the Duchenne tubules.

#### Screening for Yeast in Pickle Juice

The Jerusalem artichoke pickle juice was pretreated, diluted to different concentrations, plated onto PDA medium, labeled, and incubated at 30°C for 2–3 d to observe culture growth. The best culture concentrations were selected, and the selected strains were lined on sterilized PDA medium and cultured at 30°C for 2–3 d. The growth states were compared and observed on the medium. The suspected strains were selected and lined on the medium to isolate single colonies. The purified single colonies were cultured on chloramphenicol-supplemented YPD, Czapek-Dox Medium, and Malt Extract Agar medium at 28°C for 3–4 d to observe growth. The suspected strains were isolated and purified on Malt Extract Agar medium and stored in glycerol for later use (Liu et al., 2018). Primers against D1/D2 region of the 26S rDNA were designed, and PCR was performed to identify yeast based on the amplification.

#### Procedure for Microbial Powder Preparation

The screened and purified strains were cultured for 48 h and centrifuged at 8,000 rpm for 5 min, after which the supernatant was discarded. The collected sediment was added to the protective agent in proportion and mixed using an oscillator to prepare the microbial suspension. The suspension was then placed in the freezer, pre-cooled at 4°C for 30 min, transferred to the −30°C refrigerator for 60 min, placed in a −80°C refrigerator for 60 min, and freeze-dried for 14 h in a freeze-vacuum drying machine (the temperature of the freeze-drying chamber was −60 to −70°C with a pressure of 0.1 Pa). The powder was sealed and stored in a refrigerator at 4°C, with 10% skim milk as the protective agent.

### Brewing of High-Quality Artichoke Pickles

Curing after inulinase inactivation was performed as follows: the selected dominant microbial powder was obtained by screening and purifying the natural pickle juice. A solution of 10% (w/w) salt was added to the jar containing Jerusalem artichoke pieces with inactivated inulinase. The brine seeped out after 1–2 d, and 2% (w/w) salt was added until the top layer of the Jerusalem artichoke pieces was covered. Then, the dry bacterial powder was added. During the curing process, inulin, salt, and nitrite contents were measured every 4 d.

### Analytical Methods

#### Determination of Inulin

3,5-Dinitrosalicylic acid (7.5 g), NaOH (14 g), sodium potassium tartrate (216.0 g), and sodium sulfite (6.0 g) were dissolved separately and mixed sequentially. Finally, 5.5 mL phenol was fully dissolved in 1,000 mL of distilled water, which had been pre-boiled for 10 min. The prepared 3,5-dinitrosalicylic acid colorimetry reagent (DNS) was stored in a 4°C refrigerator away from light, and the solution was used for a period of 1 month after allowing to stand for 5 d. Crude inulin extract (1 mL) was mixed with DNA reagent (3 mL), and incubated in a boiling water bath for 5 min to allow color development. The cooled solution was diluted to an appropriate concentration with distilled water, and the absorbance was determined at 520 nm. The reducing sugar content was calculated using a standard curve.

Crude inulin extract (2 mL) was mixed with 6% phenol solution (1 mL), and then concentrated sulfuric acid (5 mL) was added rapidly, and the solution was left to cool for 10 min and then shaken well. After allowing solution to stand at room temperature for 20 min, the absorbance was measured at 490 nm. The total sugar content was calculated using a standard curve, and the inulin content was equal to the total sugar content minus the reducing sugar content.

#### Determination of Inulinase Activity

Crude inulinase solution (1 mL) was reacted with of 2% inulin (4 mL) prepared in an acetate buffer (pH 4.5) in a constant-temperature water bath (set at 55°C) for 30 min, and the reaction was terminated immediately by shifting the contents to a boiling water bath for 5 min. Pre-inactivated inulinase was used as a blank. DNS was used to measure reducing sugar content, and 1 mL of the sample was thoroughly mixed with 3 mL DNS reagent, and incubated in a boiling water bath for 5 min to allow color development. Then, the solution was diluted with distilled water to an appropriate concentration, and the absorbance was measured at a wavelength of 520 nm.

Under certain conditions, enzyme activity (U/mL) was defined as the amount of enzyme required to hydrolyze substrates to 1 g of fructose/min in a volume of 1 mL and calculated as follows.

$$\begin{array}{l} E=\frac{1000\times C\times N}{T\times V} \end{array}$$

E, inulinase activity (U/mL); C, fructose content (mg/mL) corresponding to the average absorption value of the sample aligned with the standard curve; N, dilution of crude inulinase solution; T, reaction time (min); V, crude enzyme volume involved in the reaction (mL).

#### Detection of Salt Content

Samples (5–10 g) were added to a 250 mL volumetric bottle, to which 50 mL distilled water, 1 mL potassium chromite (50 g/L), and 25 mL sample liquid were added. Another conical bottle was used as a blank control, wherein instead of the sample, distilled water was used. A silver nitrate standard solution (0.1 mol/L) was titrated to the end point (orange red), and the volume of consumption of the silver nitrate standard solution was recorded.

$$\begin{array}{l} \text{X}=\;\left(\text{V}1-\text{V}0\right)\times \text{C}\times 0.0585/\left(\text{m}\times 25/250\right)\times \text{1}00\% \end{array}$$

X, content of salt (NaCl) in the sample (%); V1, dilution of the sample used for determination of silver nitrate volume (mL) with the standard titration solution; V0, blank control of the volume of the standard titrated solution of consumed silver nitrate (mL); C, concentration of the standard titrated solution of silver nitrate (mol/L); M, mass of sample (g); 0.0585, the mass of sodium chloride that is equivalent to 1.00 mL of the silver nitrate standard solution (g).

#### Detection of Nitrite in the Samples

Pickle samples in the juicer were squeezed into a paste, and 100 g of the pickle paste was placed in the middle of a 250 mL beaker. The pickle paste—along with distilled water (80°C)—was placed in a 250 mL volumetric flask, and 2 g of carbon powder was added to remove the pigment and organic matter. Potassium ferrocyanide (2 mL) and zinc sulfate (2 mL) were used to precipitate proteins. Finally, distilled water was used to measure the volume, and a colorless transparent solution was obtained by vacuum pump filtration.

The filtrate (40 mL) was weighed and poured into a 50 mL volumetric flask, and 2 mL of paminophenesulfonic acid was added. After allowing to stand for 3 min, 1 mL naphthalene ethylenediamine hydrochloride was added, and distilled water was added to the scale. The solution was shaken and allowed to stand for 15 min, and then, the absorbance was determined at a wavelength of 540 nm using distilled water as the reference. Finally, the nitrite content in the pickles was calculated using a standard curve according.

#### Sensory Evaluation

Sensory evaluation of the Jerusalem artichoke pickles with inactivated inulinase was conducted, and the criteria and scoring matrices are shown in Table 1. The quality —measured based on taste, flavor, and color—varied depending on the manufacturing process. Here, the score was mainly based on color, flavor, and crispness, with 5 point score for each, as shown in Table 1 (de Matos et al., 2019). Sixty members of the laboratory and related major students were selected for the test, and the total score was calculated as follows: total score = color × 30% + crispness × 30% + fragrance × 40%.

**Table 1:** Sense grade of Jerusalem artichoke pickles with inulinase inactivation and microbial powder.

**Scores**	**Items**		
	**Color and Iustre**	**Fragrance**	**Brittleness**
4.5~5.0	Bright yellow, glossy without browning	Heavy flavor	Very crispy
4.0~4.5	Light yellow without obvious tan	Intense flavor	Crispy
3.5~4.0	Pale brownish yellow with partial browning of the epidermis	Light fragrance	Brittle
3.0~3.5	Epidermis of the plant with obvious browning	None	Soft
<3.0	All brown and black	Taint	Rot

## Results and Discussion

### Comparison of Inulin Content and Inulinase Activity in Jerusalem Artichokes From Different Habitats

The inulin content and inulinase activity of artichokes from different habitats, SX, CQ, XZ, YTH, and YDT, are shown in Table 2. Depending on the region of origin, the outer skin of the tuber was either red, yellow, or white; the shape was an irregular tumor or spindle; and the roots were fibrous, as shown in Figure 1. In the Jerusalem artichokes from different production areas, inulin content also differed due to different soil, water, light, temperature, and wind conditions and other external factors (de Matos et al., 2019; Lv et al., 2019). Therefore, in order to obtain higher quality Jerusalem artichoke pickles with inulin—more conducive to human health—the total sugar and reducing sugar contents of Jerusalem artichokes from SX, CQ, XZ, YTH, and YDT were measured to calculate the inulin content. The inulin content of Jerusalem artichokes ranged from 50 to 70%, which is close to the values published in literature (Lv et al., 2019). The inulin content was the highest in artichokes from SX—accounting for 68.85% of the dry Jerusalem artichoke weight—while those from YDT had the lowest inulin content (53.31%).

**Table 2:** Comparison of inulin content and inulinase activity in Jerusalem artichokes from different habitats.

	**SX**	**CQ**	**XZ**	**YTH**	**YDT**
Inulin content (%)	68.85	67.08	64.02	62.93	53.31
Inulinase (U/mL)	5.17	3.98	4.02	4.51	4.45

Further, the Jerusalem artichokes from SX exhibited the highest inulinase activity (5.17 U/mL), followed by those from YTH and YDT. The inulinase activity of Jerusalem artichokes harvested from XZ and CQ was only slightly lower than that of artichokes from other regions. Plants secrete far lower amounts of inulinase than microorganisms (Yuan et al., 2012; Singh et al., 2020). For instance, optimization of the parameters for inulinase production during *Rhizopus oryzae*-mediated fermentation using a statistical approach, resulted in maximum inulinase activity and specific activity of 348.36 EU/mL and 3621.78 EU/mg, respectively (Yazici et al., 2020).

### Effect of Different Inactivation Processes on Inulin Content and Inulinase Activity

Jerusalem artichoke inulinase is a biocatalyst regulating inulin turnover, resulting in the production of low poly fructose or high-fructose syrup. However, inulin hydrolysis by inulinase should be avoided; as loss of inulin would result in low-quality Jerusalem artichoke pickles. The inulinase activity of Jerusalem artichokes can be affected, to different degrees, by the physical and chemical properties of Jerusalem artichokes, the microorganisms present during the pickling process, and human intervention.

#### Salt Stress

**Figure 2:**
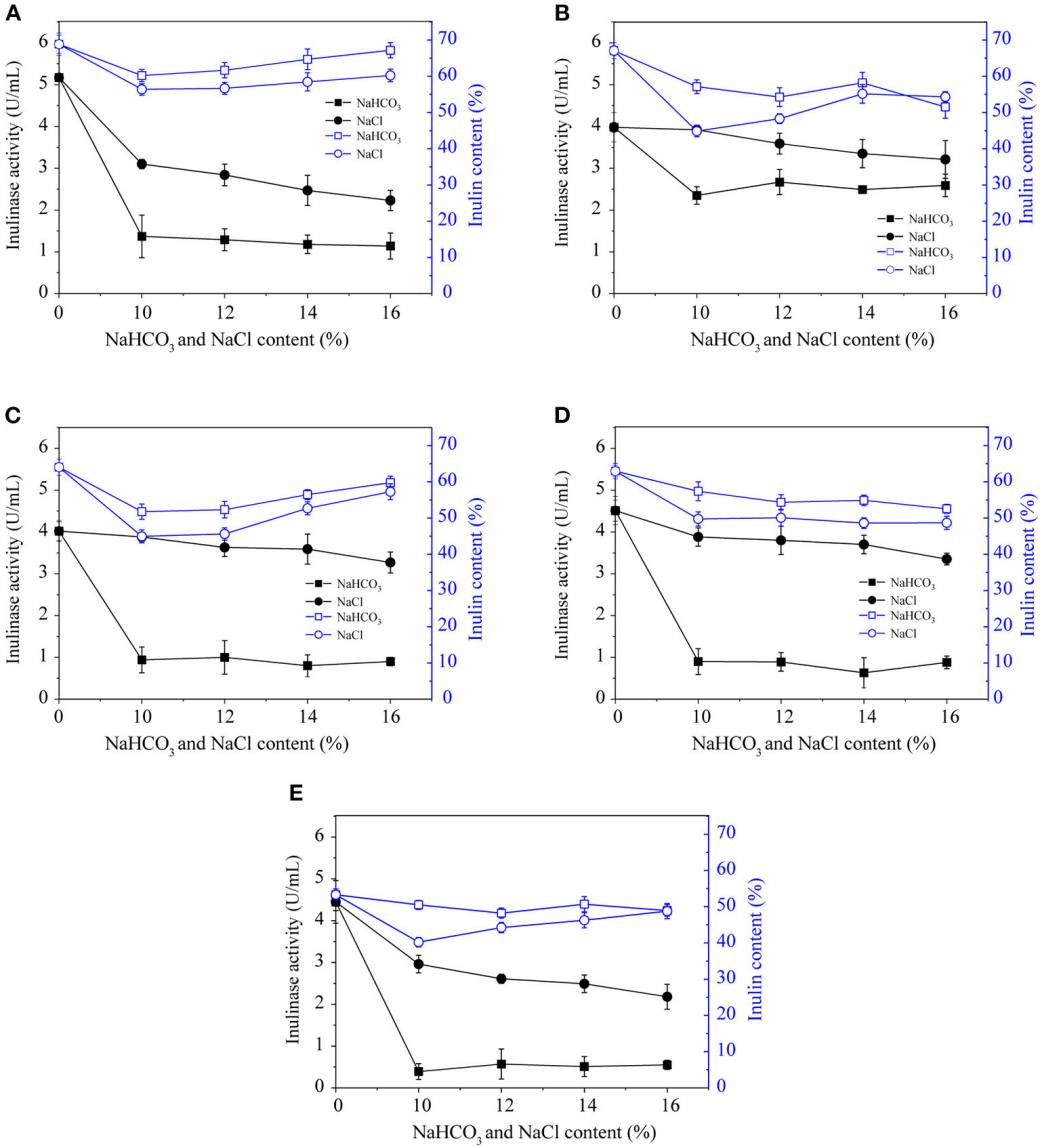
The effects of NaHCO_3_ and NaCl on the inulinase activity and inulin content of Jerusalem artichoke tubers from SX **(A)**, CQ **(B)**, XZ **(C)**, YTH **(D)**, and YDT **(E)**.

NaCl is an abiotic stress that reduces the inulinase activity during pickling process of Jerusalem artichoke. The soil environment, osmotic stress, and ion poisoning due to salt could also inhibit inulinase activity. As shown in Figure 2, a solution of 10–16% NaCl was used to inactivate inulinase during the pickling of Jerusalem artichokes. Higher salt concentrations were correlated with greater ability to inactivate the enzyme, but the variation in inulinase activity was limited among samples treated with varying concentrations of NaCl (10–16%) according to Figure 2. Therefore, in response to the increasing adaption of low-salt lifestyles, 10–12% NaCl is considered to be good choice for inactivating inulinase in Jerusalem artichokes. When NaCl was used to inactivate inulinase, the artichokes initially exhibited a flaxen appearance; only slight browning and softening occurred over time, and the flavor was considerable (Luo et al., 2018). According to feedback from the Jerusalem artichoke brewing plant, the Jerusalem artichoke acquired after maturation can be washed and pickled with only 10% NaCl in the middle of October every year. This might be due to the low temperature in winter, leading to reduced activity of various mixed bacteria compared with that in spring and summer (Li et al., 2015); thus, less salt is used.

#### Treatment With NaHCO_3_

NaHCO_3_ has been widely used in analytical, synthetic, pharmaceutical, medical, and food fermentation. It does not alter the dry weight of the cells in the fermentation system during vegetable curing, but it significantly increases the rate of glucose consumption (Zhang et al., 2019). According to Figure 2, a 10–16% solution of NaHCO_3_ can inactivate inulinase during Jerusalem artichoke pickling. The inulinase activity of Jerusalem artichokes from CQ, after NaHCO_3_ treatment, was ~2.5 U/mL. After Na_2_HCO_3_ treatment, the inulinase activity of Jerusalem artichokes from the remaining four regions was similar, ~0.5–1.5 U/mL. However, the Jerusalem artichoke tubers exhibited browning and became soft during marination. Furthermore, artichoke appearance, taste, and sensory qualities were unsatisfactory when inactivation was performed using NaHCO_3_. In conclusion, Jerusalem artichoke tubers were damaged by NaHCO_3_ stress to a much greater extent than they were in response to NaCl stress, as evidenced by the oxidative damage in mulberry seedling leaves (Zhang et al., 2019, 2020).

#### Ultrasonic Treatment

**Figure 3:**
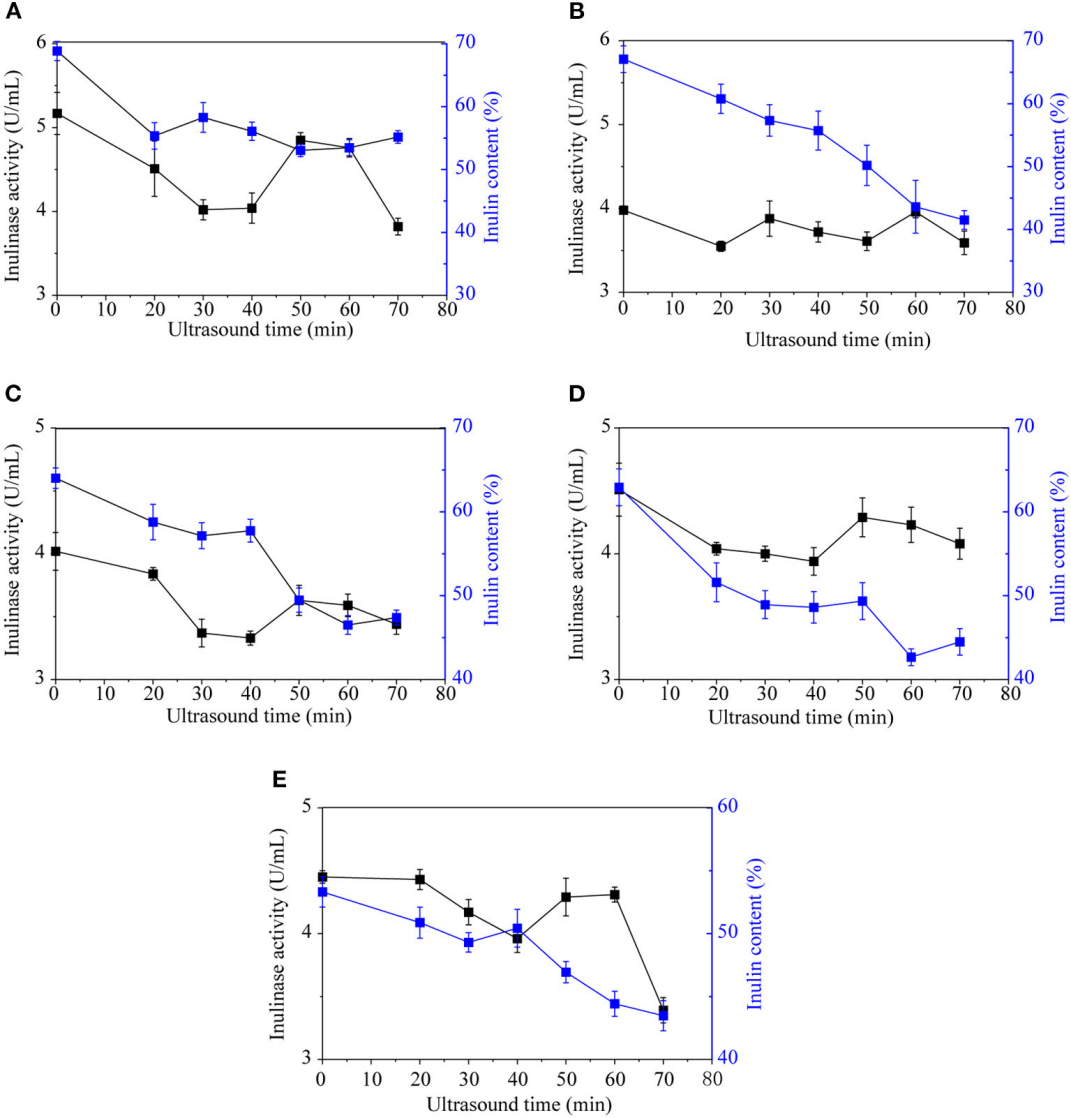
The effect of ultrasound on the inulinase activity and inulin content of Jerusalem artichoke tubers from SX **(A)**, CQ **(B)**, XZ **(C)**, YTH **(D)**, and YDT **(E)**.

Ultrasonic waves are mechanical vibrations that can alter or accelerate changes in material performance, state, structure, and organization (Soria and Villamiel, 2010; Shoaib et al., 2016). Currently, ultrasonic processing is widely used as a new non-thermal technology for enzyme inactivation. Jerusalem artichoke inulinase could be inactivated by ultrasound, with an optimum effect at an ultrasonic time of 30–40 min (Figure 3). Ultrasonic treatment was most effective against inulinase from Jerusalem artichoke tubers from SX, with the inulinase activity decreasing from 5.17 to 4.02 U/mL. When the ultrasonication time was extended to 50–60 min, enzyme activity increased slightly because the temperature approached the optimum temperature (55°C) required by Jerusalem artichoke inulinase for its activity. As the ultrasonic time continues to extend, high levels of energy are generated through extremely high-density shock waves, leading to extreme physical effects such as high temperature, high pressure, etc. This may alter the molecular structure and conformation of inulin, and enzyme activity might be reduced by the strong shear force and shock waves.

Ultrasound can damage the activity of enzymes, thereby affecting normal cells as well as energy metabolism, and even leading to cell death. Several studies have shown that the activity of ATP and catalase decreased after ultrasonic treatment, and ultrasonic treatment has also enhanced enzymatic browning (Soria and Villamiel, 2010). Various effects of ultrasonic waves, such as increased temperature, increased pressure, and vibration, can accelerate molecular movement, thus accelerating inulinase secretion into the medium and promoting the hydrolysis of Jerusalem artichoke inulin. The Jerusalem artichoke tubers from different regions are irregular, with corners unwashed. Ultrasound can be used to deeply clean Jerusalem artichokes and inactivate inulinase, and it can be integrated with PPO enzyme inactivation, thereby reducing browning in the pickling process.

#### Combination of Salt and Ultrasonic Treatment

As shown in Figure 2, NaHCO_3_ or salt stress was able to inactivate the inulinase of Jerusalem artichokes. NaHCO_3_ achieved significantly better enzyme inactivation and inulin levels than NaCl. Further, 10–16% NaHCO_3_ exhibited potent inulinase inactivating ability but resulted in bad flavor; thus, this treatment is not suitable for inactivating inulinase during the pickling of Jerusalem artichokes. In conditions of salt stress, higher concentrations of NaCl are correlated with a greater degree of enzyme inactivation and improved sensory qualities of Jerusalem artichokes. Under room temperature conditions, the frequency of the ultrasonic cleaner was fixed at 40 kHz, the ultrasonic power at 480 W, and the ultrasonic treatment at 30 min for Jerusalem artichokes purchased from SX. Then, 10% NaCl was added for enzyme inactivation. The Jerusalem artichoke with the highest inulin content was selected, and the inulinase was inactivated via ultrasound and NaCl. In response to this combined treatment, the inulinase activity was reduced to 2.50 U/mL, and the inulin percentage was 61.22%. Therefore, this strategy of using a combination of ultrasound and NaCl to inactivate inulinase could preserve the inulin content of Jerusalem artichokes during curing and maintain the beneficial health effects of these pickles (Shoaib et al., 2016; Yu et al., 2018).

### Preparation of Microbial Powder

#### Physiological, Biochemical, and Molecular Identification of Suspected Strains

In accordance with procedure 2.3, eight suspected *Lactobacillus* strains (labeled LS1-8) and seven yeast strains (labeled YS1-7) were selected from the pickle juice samples obtained from pickle factories as experimental strains. The eight suspected *Lactobacillus* strains were gram-positive. LB medium was used in the anaerobic test tube, and 0.1% CaCO_3_ was added to solidify the sample. LS3 and LS6 grew well under anaerobic conditions, showing milky white and generally linear growth (Contreras-Hernández et al., 2018), while the growth of LS1 and LS8 was extremely poor. Lactic acid bacteria are commonly identified in pickles because of their ability to produce high levels of lactic acid and survive under highly acidic conditions (Zokaeifar et al., 2012). Indeed, pickles fermented by lactic acid bacteria have a distinctive flavor and exhibit positive effects on health (Irkin and Songun, 2012). The seven suspected yeast strains were molded into water immersion tablets and observed under a microscope. YS1 grew slightly worse, while YS2 and YS5 grew well compared with the other strains. The surface of the colony was white and smooth, the individual shape of the species was spherical or elliptical, and the flavor was strong.

#### Growth Curves of Microbial Species Under Different Salinity Conditions

**Figure 4:**
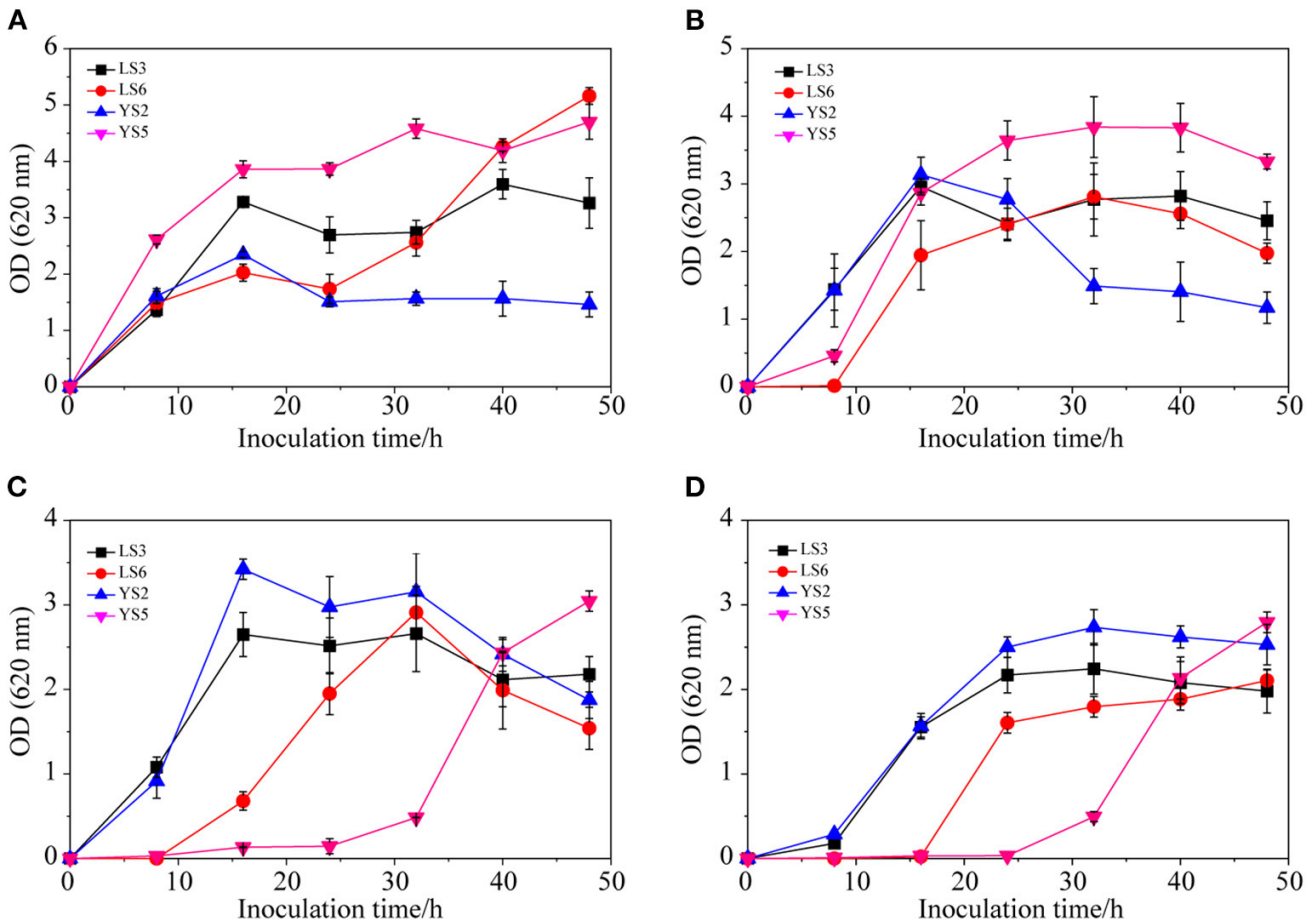
Growth curves of different strains in the presence of 1% NaCl **(A)**, 4% NaCl **(B)**, 7% NaCl **(C)**, and 10% NaCl **(D)**.

Of the 15 strains (LS1-8 and YS1-7), four strains (LS3, LS6, YS2, and YS5) with good growth status were selected for salt tolerance experiments. As shown in Figure 4A, in response to 1% NaCl, the growth of the four strains was relatively fast initially and then flattened or decreased over 16 h. When the NaCl concentration was increased to 4% (Figure 4B), the growth of LS3 (OD_620_ = 2.96) and YS2 (OD_620_ = 3.14) was relatively fast in the first 16 h. After 16 h, LS6 (OD_620_ = 1.95) and YS5 (OD_620_ = 2.87) still exhibited slow growth that did not decline until 32 h later. When the NaCl concentration increased to 7% (Figure 4C), the growth of LS3 (OD_620_ = 2.65) and YS2 (OD_620_ = 3.42) was faster within the first 16 h, but flattened or declined after 16 h, while LS6 and YS5 grew slowly within the first 8 h. The growth of all four strains in the first 8 h was rather slow in the presence of high salinity (10% NaCl) (Figure 4D). LS3 and YS2 exhibited a higher growth rate from 8 to 32 h, indicating that LS3 and YS2 were more resilient to 10% NaCl and could maintain high density for a long time. To summarize, YS2 and LS3 exhibited greater salt tolerance compared with strains YS5 and LS6, which could adapt to the salt concentration over a short time as the flora reproduced, thereby inhibiting the growth of other hybrid strains. Therefore, yeast YS2 and lactic acid bacteria LS3 were found to be Jerusalem artichoke brewing, and a dry powder made of YS2 and LS3, according to procedure 2.3.3, was added into the curing pot to reduce the curing period.

### Alterations in Jerusalem Artichoke Pickle Components in Response to Different Salt Concentrations With Screened Strains

In contrast to the traditional pickling methods, in this study, we employed the strategy of adding a dry powder composed of the dominant strains YS2 and LS3 during the curing process, and measured the inulin and nitrite contents.

**Figure 5:**
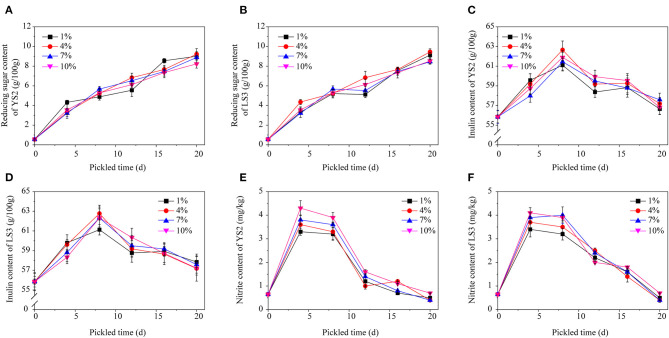
The change in the content of reducing sugar, inulin, and nitrite in response to different salt concentrations. **(A)** Reducing sugar of YS2, **(B)** reducing sugar of LS3, **(C)** inulin content of YS2, **(D)** inulin content of LS3, **(E)** nitrite content of YS2, **(F)** nitrite content of LS3.

Microbial powders made of YS2 and LS3 were independently added to four salt concentrations (1, 4, 7, and 10%). The change in reducing sugar content is shown in Figures 5A,B. Initially, the reducing sugar content of the Jerusalem artichokes was low (0.78/100 g), but it gradually increased to 9.42/100 g. There was no significant change in the reducing sugar content between the two strains or between different salt concentrations. The reason for the gradual increase in the reducing sugar content was that the high salt concentration outside of the Jerusalem artichokes caused loss of water from the artichokes, resulting in the hydrolysis of polysaccharides into monosaccharides.

The change in inulin content is an important index to ensure the curing quality of Jerusalem artichokes. The inulin content exhibited an upward trend in the first 8 d and a downward trend until 12 d (Figures 5C,D). After curing for ~20 d, the inulin generally decreased to ~55/100 g. The trend of increasing inulin concentrations during the early stage may be attributes to the loss of water from Jerusalem artichoke tissues, while the decrease in inulin content in the later stage indicates inulin partially degradation.

It can be seen from Figures 5E,F that the nitrite content on the fourth day was relatively high, and even exceeded the national standard of 4 mg/kg, which was higher than the initial nitrite content. In the early pickling stage, nutrients oxidize the nitrate in vegetables into nitrite via the action of microorganisms. At the same time, phenolic compounds in vegetables also reduce nitrite; however, the degree of nitrite generation is much higher than the degree of nitrite reduction, so the nitrite content in the early stage increases dramatically. However, the salts and sugar present in Jerusalem artichokes inhibit the growth of microorganisms as well as the consumption of oxygen. Meanwhile, the nitrate content in vegetables reduces owing to oxidation, so the nitrite level drops to a relatively stable value (Hou et al., 2013; Kandasamy et al., 2018).

The 16S rDNA universal primer was designed to identify LS3, and the whole genomic DNA of strain LWFQ2048 was used as the template. A target fragment of ~1,468 bp was obtained, which was purified and sent for sequencing. The sequencing results were used to construct phylogenetic trees using SPSS data analysis software for molecular identification, as shown in Supplemental Figure 1, and the strain LS3 was further identified as *Enterococcus faecalis*. Primers against D1/D2 region of the 26S rDNA were designed, and PCR was performed to identify strain YS2 based on the amplification of a target fragment of ~538 bp. The sequencing results were used to construct phylogenetic trees using SPSS data analysis software for molecular identification, as shown in Supplemental Figure 2, and the strain YS2 was further determined to be the salt-tolerant yeast *Meyerozyma guilliermondii*. The isolation of more lactic acid bacterial and yeast strains from the pickling juice has been under investigation. It's possible to develop a defined mixed culture for the pickling process based on the in-depth study.

In this study, sixty members of the laboratory and related major students were selected for sample evaluation. The total score was calculated as: total score = color × 30% + crispness × 30% + fragrance × 40%. The taste of the Jerusalem artichoke pickles significantly improved after adding LS3 and YS2 microbial powders. Notably, the sensory evaluation score of inulinase-inactivated Jerusalem artichoke pickles tubers with the microbial powder was 4.15 ± 0.27 (color was 4.03 ± 0.42, crispness was 4.18 ± 0.36, and fragrance was 4.23 ± 0.36), whereas that of naturally pickled artichokes was 3.75 ± 0.36 (color was 3.60 ± 0.48, crispness was 3.79 ± 0.47, and fragrance was 3.83 ± 0.68), and significance (*p* < 0.001) was evaluated by SPSS. The new method greatly shortens the fermentation cycle, reduces the amount of salt, and ensures that the nitrite content is lower than the national standard.

## Conclusions

In this study, we identified a feasible strategy for inactivating of Jerusalem artichoke inulinase. The inulinase in Jerusalem artichoke pickles was inactivated by employing a combination of salt stress and ultrasonic treatment, without any pronounced deterioration or color changes. Ultrasound for 30–40 min combined with 10% NaCl was found to be the best treatment to effectively deactivate inulinase while still producing a high-quality and flavorful pickle. Furthermore, two Jerusalem artichoke pickling strains were identified. A dry powder composed of lactic acid bacteria and yeast strain isolated from pickle juice samples obtained from a pickle factory were used to pickle Jerusalem artichokes with inactivated inulinase, which reduced the fermentation time to ~20–30 d and improved the quality of the final product.

## Data Availability Statement

The original contributions presented in the study are included in the article/Supplementary Material, further inquiries can be directed to the corresponding author.

## Author Contributions

All authors listed have made a substantial, direct and intellectual contribution to the work, and approved it for publication.

## Conflict of Interest

The authors declare that the research was conducted in the absence of any commercial or financial relationships that could be construed as a potential conflict of interest.
