# Supplementary material for: Inactivation of Inulinase and Marination of High-Quality Jerusalem Artichoke (Helianthus tuberosus L.) Pickles With Screened Dominant Strains
Source: Front Bioeng Biotechnol. 2021 Jan 20;8:626861. doi: 10.3389/fbioe.2020.626861 (PMC7855583; doi:10.3389/fbioe.2020.626861)
Supplement: Supplementary file 1 [file Data_Sheet_1.DOCX]

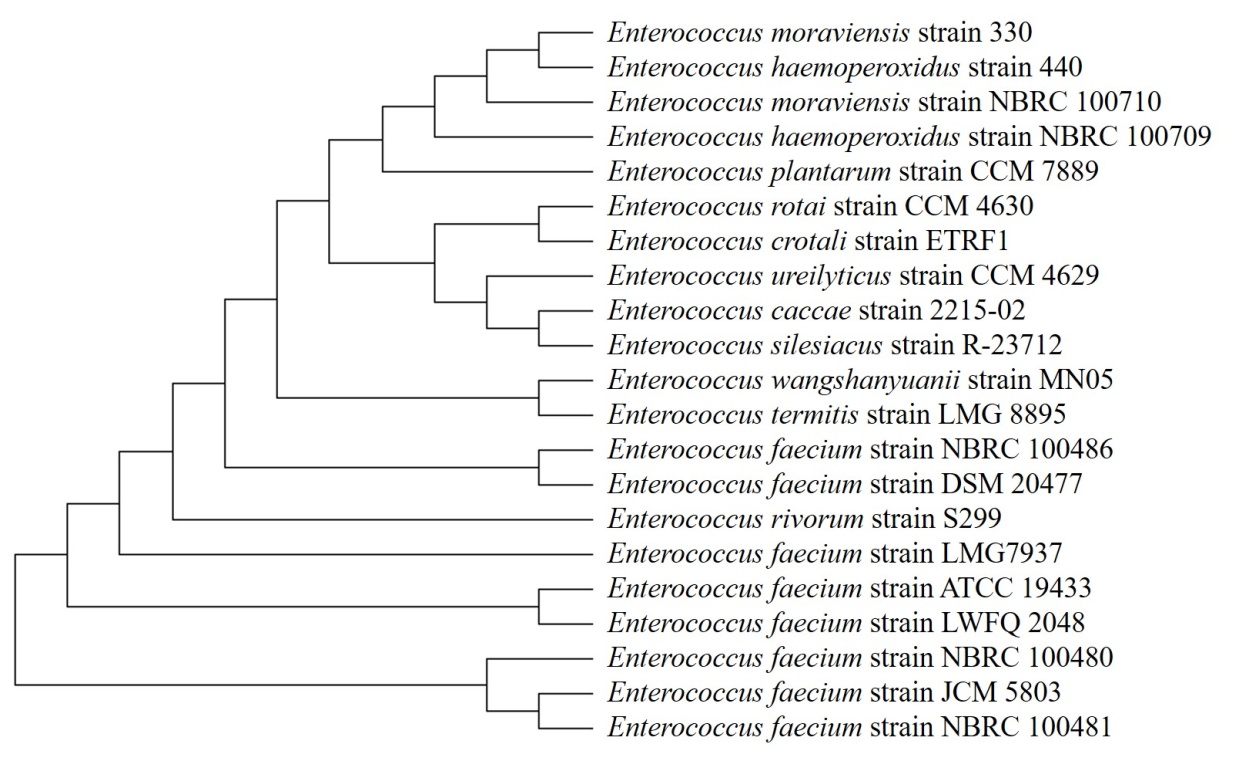


**Supplemental Fig.1 Phylogenetic tree of the dominant strain LS3**


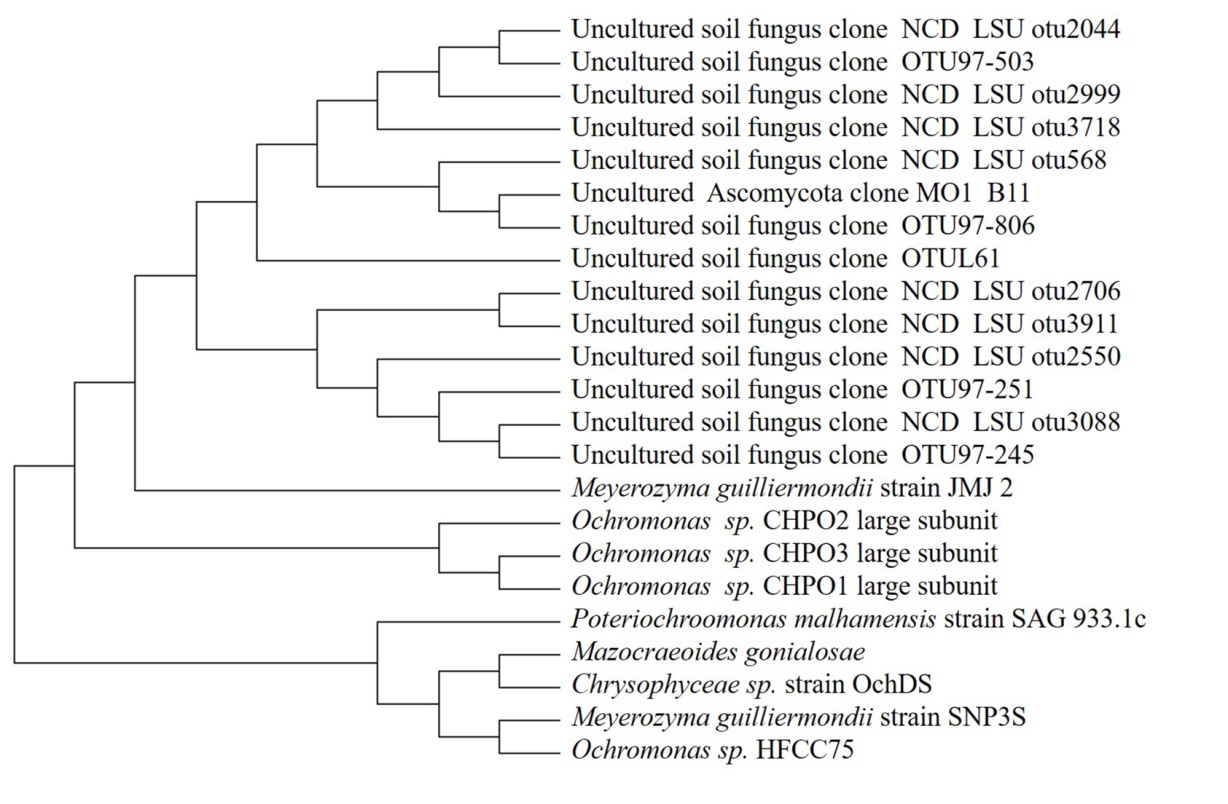


**Supplemental Fig.2 Phylogenetic tree of the dominant strain YS2**
